# Increase of Soluble RAGE in Cerebrospinal Fluid following Subarachnoid Haemorrhage

**DOI:** 10.1155/2017/7931534

**Published:** 2017-05-29

**Authors:** Bartosz Sokół, Norbert Wąsik, Roman Jankowski, Marcin Hołysz, Witold Mańko, Robert Juszkat, Tomasz Małkiewicz, Paweł P. Jagodziński

**Affiliations:** ^1^Department of Neurosurgery, Poznan University of Medical Sciences, Poznan, Poland; ^2^Department of Biochemistry and Molecular Biology, Poznan University of Medical Sciences, Poznan, Poland; ^3^Department of Anaesthesiology and Intensive Therapy, Poznan University of Medical Sciences, Poznan, Poland; ^4^Department of General and Interventional Radiology, Poznan University of Medical Sciences, Poznan, Poland

## Abstract

Receptors for advanced glycation end-products (RAGE) mediate the inflammatory reaction that follows aneurysmal subarachnoid haemorrhage. Soluble RAGE (sRAGE) may function as a decoy receptor. The significance of this endogenous anti-inflammatory mechanism in subarachnoid haemorrhage (SAH) remains unknown. The present study aims to analyse sRAGE levels in the cerebrospinal fluid (CSF) of SAH patients. sRAGE levels were assayed by ELISA kit in 47 CSF samples collected on post-SAH days 0–3, 5–7, and 10–14 from 27 SAH patients with acute hydrocephalus. CSF levels of sRAGE were compared with a control group and correlated with other monitored parameters. In the control group, the CSF contained only a trace amount of sRAGE. By contrast, the CSF of 20 SAH patients collected on post-SAH days 0–3 was found to contain statistically significant higher levels of sRAGE (mean concentration 3.91 pg/mL, *p* < 0.001). The most pronounced difference in CSF sRAGE levels between good and poor outcome patients was found on days 0–3 post-SAH but did not reach the significance threshold (*p* = 0.234). CSF sRAGE levels did not change significantly during hospitalisation (*p* = 0.868) and correlated poorly with treatment outcome, systemic inflammatory markers, and other monitored parameters. Our study revealed an early and constant increase of sRAGE level in the CSF of SAH patients.

## 1. Introduction

The management of aneurysmal subarachnoid haemorrhage (SAH) has seen no significant advance since the introduction of nimodipine and the use of Guglielmi detachable coils [1, 2]. Favourable outcomes occur in only about one-third of patients admitted in a poor neurological state, and no new drugs have been approved for use in SAH in the past two decades; hence there is an urgent need to look for new therapies [3–8]. Early brain injury (EBI) is considered a promising target for future research [9, 10]. This represents the pathophysiological events occurring during the first 72 h following SAH and strongly determines the mortality and morbidity [11]. Experimental models support a number of mechanisms for EBI including inflammation [9]. Further clinical studies need to determine which of these mechanisms predominate. The present writers regard inflammation as a promising target for investigation. On a cellular level, inflammation is triggered by a ligand-receptor interaction. Among the most abundant multiligand are receptors for advanced glycation end-products (RAGE). RAGE has been shown to be present in neurons, glia, and microglia in the human hippocampus and cortex [12]. The concentration of many of its ligands (e.g., high mobility group box 1 protein, S100B protein) in plasma or cerebrospinal fluid (CSF) correlates with the clinical outcomes in patients with SAH [13–15]. Binding of these ligands to RAGE leads to the recruitment of multiple intracellular signalling molecules and eventually activates pathways responsible for acute and chronic inflammation [16]. There is a growing body of evidence that RAGE and its ligands are involved in the pathogenesis of other disorders including some cardiovascular conditions, neurodegenerative processes, and autoimmune diseases [17]. The soluble isoform of RAGE (sRAGE) corresponds to the extracellular domain of RAGE lacking cytosolic and transmembrane domains. As a decoy receptor, sRAGE is able to bind the same ligands as a membrane-bound form but unable to trigger the intracellular responses. The anti-inflammatory potential was confirmed in a mouse experimental stroke model, where intravenous administration of recombinant sRAGE significantly reduced infarct size and improved functional outcome [18]. The soluble form of RAGE has also been widely recognised as a biomarker. CSF levels of sRAGE were observed to be reduced in Guillain-Barré syndrome and multiple sclerosis [19, 20]. In view of the presence of sRAGE in CSF, its significant role in ischaemic stroke, and the role of its ligands in SAH, we aim to analyse sRAGE levels in CSF of patients with SAH requiring acute treatment of hydrocephalus.

## 2. Materials and Methods

### 2.1. Study Population

This single-centre, observational, prospective study was conducted in accordance with the Declaration of Helsinki and its protocol was approved by the local bioethics committee. Between January 2015 and September 2016, twenty-seven patients met the enrollment criteria which are as follows: (1) SAH confirmed by computed tomography (CT), (2) early (<24 h) endovascular treatment, (3) acute hydrocephalus diagnosed on CT and managed with external ventricular drainage (EVD) < 48 h, and (4) informed consent (by patient or family). Patients below the age of 18 were excluded due to physiological differences in CSF content as well as distinct aSAH presentation, aneurysm morphology, and outcome [21, 22]. Also excluded were patients with central nervous system (CNS) disease and those with active systemic diseases (diabetes mellitus, rheumatoid arthritis, malignancy, cirrhosis, and renal failure). Meticulous care was taken to rule out patients with signs of EVD infection. CSF cell count was checked at least twice per patient and CSF culture was ordered at least once on post-SAH days 10–14. SAH management in our unit involves the continuous intravenous infusion of nimodipine for at least ten days, whilst avoiding hypotension by means of vasopressors. CT scan of the head was carried out at least twice in every patient: on post-SAH days 2-3 to assess procedure related injury and before discharge to assess delayed cerebral ischaemia. The control samples of CSF were obtained during anaesthesia from 20 patients with a negative history of CNS disease.

### 2.2. End Points

Subjects were followed until death or the completion of 3 months following SAH. The primary outcome was the functional state after 3 months, and the secondary outcome was in-hospital mortality. The functional outcome was defined using the Glasgow Outcome Scale (GOS); these were dichotomized as good (GOS 4-5) or poor (GOS 1–3) outcomes.

### 2.3. Sample Collection and Assays

CSF samples were collected from the EVD at three time points, on post-SAH days 0–3, 5–7, and 10–14. The final sRAGE assays comprised twenty samples from days 0–3, sixteen from days 5–7, and eleven from days 10–14. A complete set of three samples was obtained from only five patients on account of suspected EVD infection, EVD obstruction, or early EVD removal. Each sample was centrifuged and stored at −80°C until assayed. The sRAGE assays were carried out using ELISA commercial kit RAB0007-1KT (Sigma Aldrich, St. Louis, USA). The minimum detectable level of sRAGE was 2.06 pg/mL, and linearity was conserved between 2.06 pg/mL and 1500 pg/mL. Haemoglobin (Hgb) level, C-reactive protein (CRP) level, and white blood cell (WBC) count and fibrinogen level were assessed daily by automatic analysers XT 2000i (Sysmex, Japan), Cobas 6000 (Roche Diagnostic, USA), and ACL TOP 500 (Instrumentation Laboratory, Italy).

### 2.4. Statistical Analysis

In the tables, values for numerical data have been expressed as mean and standard deviations; for ordinal numerical data, they are expressed as median and interquartile range and for categorical data as counts and percentages. In the figures, all data are presented as mean and standard deviations. The normality of data distribution was assessed using the Shapiro-Wilk test. The correlations were assessed by Spearman's test, and correlation coefficient (cc) > 0.6  (cc < −0.6) was considered significant. A value of *p* < 0.05 was considered statistically significant when comparing. Data were analysed using Statistica 10 (Statsoft, Inc., Tulsa, OK, USA).

## 3. Results

**Figure 1:**
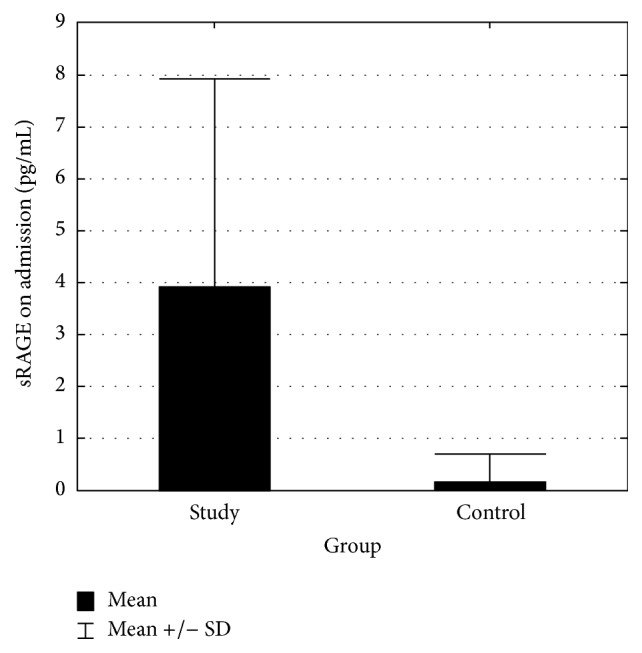
sRAGE level in study and control group. Mann–Whitney test revealed significantly higher (*p* < 0.001) levels of sRAGE in CSF of SAH patients.

**Figure 2:**
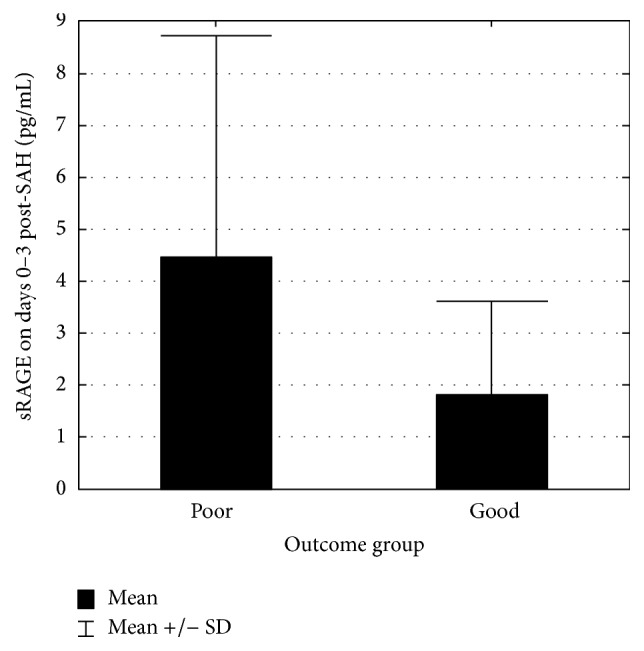
sRAGE level in patients with poor and good treatment outcome on days 0–3 post-SAH. Mann–Whitney test revealed no significant difference (*p* = 0.234) in sRAGE level between patients with good and poor treatment outcome.

**Figure 3:**
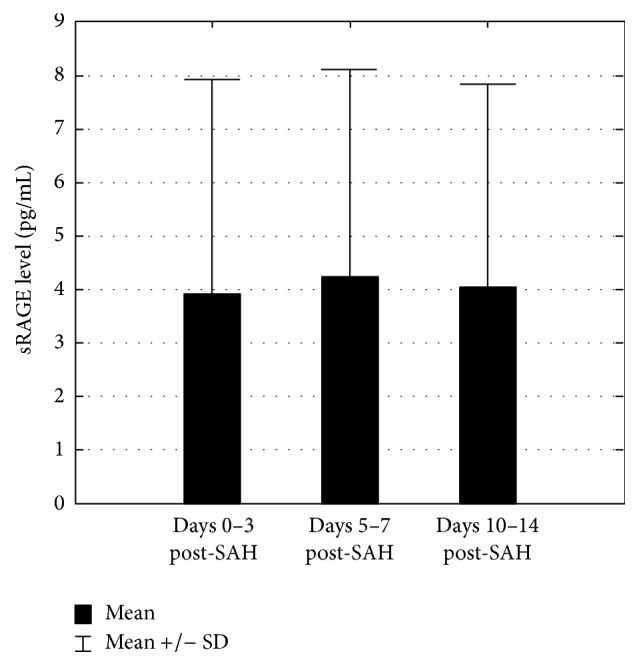
sRAGE level changes in time in study group. Skillings-Mack test indicates no significant change of CSF sRAGE level during hospitalisation (*p* = 0.868).

The control group consisted of twenty-five patients free of CNS disease. Detectable levels of sRAGE were found in only two members of this group (CSF sRAGE of 2.1 and 1.87 pg/mL). The details of the study group are presented in Table 1. CSF collected on days 0–3 following aneurysmal rupture in twenty of these patients contained statistically significant higher levels of sRAGE (*p* < 0.001) (Figure 1). No sRAGE was found in four of these twenty. Mean concentration varied significantly (0–15.22 pg/mL) but failed to differentiate good and poor outcome. The most pronounced difference between good and poor outcome was found at this stage but did not achieve statistical significance (*p* = 0.234) (Figure 2). The *p* values for days 5–7 and 10–14 were 0.291 and 0.490, respectively. Furthermore, CSF sRAGE levels did not change significantly during hospitalisation (*p* = 0.868) (Figure 3). Spearman's test revealed that the strongest correlation with outcome (measured by GOS at 3 months) were the admission grades Hunt and Hess (HH) (cc = −0.656), Glasgow Coma Scale (GCS) (cc = 0.688), and World Federation of Neurosurgical Societies (WFNS) (cc = −0.741), together with the fibrinogen level on days 10–14 post-SAH (cc = −0.626). sRAGE levels, haemoglobin, and blood inflammatory markers (CRP, WBC) showed poor correlation with treatment outcome (Table 2). The sRAGE levels of patients scoring 5 on the WFNS scale at days 0–3 showed a stronger correlation (cc = 0.485) with treatment outcome than those scoring 5 on the HH scale (cc = 0.176).

## 4. Discussion

Our study demonstrated elevation of CSF sRAGE in patients with poor grade SAH requiring EVD insertion. Clinical studies of sRAGE in patients with neurological disorders have thus far revealed the following: (1) serum sRAGE elevation in ischaemic stroke patients [18], (2) serum sRAGE correlation with severity of the axonal subtype of Guillain-Barré syndrome [19], and (3) CSF sRAGE decrease in patients with multiple sclerosis and Guillain-Barré syndrome [19, 20]. We are not aware of any previous reports demonstrating elevation of sRAGE in CSF following pathological processes, particularly in patients with SAH.

RAGE is a transmembrane protein that belongs to the immunoglobulin superfamily [18, 23–25]. The human RAGE gene is located on chromosome 6 and its expression leads to production of a 55-kDa type I membrane glycoprotein [24]. Soluble isoforms of RAGE are formed either by (1) removal of the transmembrane region from the pre-RNA during alternative splicing (leading to the production of endogenous sRAGE) or (2) proteolytic cleavage of the full-length membrane form of RAGE protein (mRAGE) by a membrane metalloproteinase called ADAM 10 or an extracellular matrix metalloproteinase 9 (MMP-9) [25–27]. ADAM 10 is a representative of sheddases, membrane-bound enzymes that cleave extracellular portions of transmembrane proteins, releasing the soluble ectodomains from the cell surface. In healthy population, mean blood plasma sRAGE concentration ranges from 800 to 1500 pg/mL [28, 29]. In our study, CSF sRAGE levels in control patients without a history of neurological disorder were undetectable. This finding is in accordance with the recent findings of Zhang et al. [19]. A rat experimental SAH model revealed significant increases in RAGE protein and mRNA levels in neurons and microglia [30]. Furthermore, an increase of MMP-9 levels in both CSF and serum was observed during SAH [31]. Based on these findings, we suspect that three mechanisms are leading to an increase of sRAGE levels in the CSF in our patients. Firstly, SAH-induced expression of RAGE leads to overexpression of all its isoforms, including endogenous sRAGE. This explanation follows Tang et al. hypothesis that high levels of plasma sRAGE at 48 h after stroke may reflect the rapid activation of mRAGE expression induced by the cerebral ischaemia [18]. A second possible mechanism is an excessive cleavage of membrane-bound RAGE. Increase of RAGE expression on cell membranes and rise of MMP-9 level (both observed during SAH) support this hypothesis [30, 31]. A third expected mechanism is introduction of free plasma sRAGE during aneurysm rupture and blood extravasation to subarachnoid space. Estimated total SAH volume equals 35 mL and as a blood contains 200 to 400 times higher sRAGE levels than those measured in CSF, we would expect more significant elevation of sRAGE levels originating from the SAH patients analysed in our study [32].

As our understanding of the SAH complications has improved, identifying mediators of its critical pathways and designing new targeted therapies becomes of primary importance in neuroproteomics research [33]. The inflammatory reaction, which contributes to SAH-induced brain injury is characterised by complex, multilevel interactions between its separate components. The activation of nuclear factor kappa-light-chain-enhancer of activated B cells (NF-kB) observed in SAH leads to excessive inflammation and subsequent brain injury [34, 35]. NF-kB activation is mediated by numerous upstream pathways, including those starting at RAGE and Toll-like receptors 2 and 4 (TLR2/TLR4). Results of our previous studies on soluble TLR2 and 4 suggested that these played only a minor role in this inhibitory mechanism [36]. Tang et al. in their study of sRAGE in human stroke patients found sRAGE to be an independent predictor of functional outcome. In experimental settings, administration of recombinant sRAGE significantly improved the outcome after ischaemic stroke in mice. The suspected protective mechanism depends on high mobility group box 1 binding [18]. Quade-Lyssy et al. have reported that atorvastatin increased the levels of serum sRAGE [37], whilst Cheng et al. [38] and Potey et al. [39] have shown some evidence that atorvastatin ameliorates vasospasm and EBI after SAH. However, the STASH failed to detect any benefit to the long- or short-term outcome using simvastatin in aneurysmal SAH [40]. In the most recent report by Wang et al., administration of recombinant sRAGE significantly reduced the number of positive TUNEL staining cells in SAH rat and improved cell viability in post-SAH CSF-treated cultured neurons [41]. In our study, sRAGE levels failed to differentiate between good and poor outcome patients. These preliminary findings are similar to the results obtained with soluble TLR2/TLR4 and might suggest that sRAGE has limited significance as a prognostic biomarker. Yet, further investigations (addressing limitations of the current study) are essential to assess role of sRAGE in the endogenous anti-inflammatory mechanism. Our study showed no correlation between sRAGE and systemic inflammatory mediators. Although CRP is able to augment mRNA expression of RAGE genes, the levels to which this can be achieved are not known [42].

Conclusions are limited by the small number of patients involved in the study and lack of consecutive sampling in most cases. The enrolled patients do not represent the full spectrum of SAH as hydrocephalus was an inclusion criterion and could have contributed to brain injury before EVD insertion. Despite careful monitoring, EVD infection remains a potential bias. In addition, only a few elements of the inflammatory pathway were investigated, and further investigation will be required to elucidate the larger picture of post-SAH inflammation.

## 5. Conclusions

CSF levels of sRAGE increase early in patients with SAH who require acute treatment of hydrocephalus and remain elevated but do not correlate with treatment outcome. The significance of sRAGE as an endogenous anti-inflammatory mechanism requires further investigation.

## Figures and Tables

**Table 1:** Study group patients' characteristic.

Male	15 (56%)		
Age (years)	58.07 ± 15.8		

Aneurysm location			

Middle cerebral artery	7 (26%)		
Anterior communicating artery	7 (26%)		
Anterior cerebral artery	4 (15%)		
Basilar artery	4 (15%)		
Internal carotid artery	3 (11%)		
Posterior cerebral artery	2 (7%)		

Aneurysmal size (mm)	5.08 ± 1.8		
Cerebral infarction due to DCI on CT	20 (74%)		
Intracerebral haemorrhage on CT	14 (52%)		
Intraventricular blood on CT	26 (96%)		
Fisher CT score	4 (4-4)		
Modified Fisher CT score	4 (2–4)		
WFNS score on admission	5 (3–5)		
HH score on admission	4 (4-5)		
GCS on admission	5 (4–10)		

	Post-SAH days 0–3	Post-SAH days 5–7	Post-SAH days 10–14

CRP level (mg/L)	106.90 ± 88.9	129.68 ± 96.8	75.09 ± 84.3
WBC count (10^6^/mm^3^)	13.81 ± 5,4	12.01 ± 5.0	14.35 ± 6.3
Hgb level (mg/dL)	12.52 ± 1.7	12.10 ± 1.6	10.84 ± 1.2
Fibrinogen (mg/dL)	415.61 ± 163.0	617.63 ± 241.1	600.33 ± 247.7
sRAGE (pg/mL)	3.91 ± 4.0	4.24 ± 3.9	4.05 ± 3.8

Treatment outcome (according to GOS at 3 months)			

Low disability (score of 5)	5 (19%)		
Moderate disability (score of 4)	2 (7%)		
Severe disability (score of 3)	4 (15%)		
Persistent vegetative state (score of 2)	5 (19%)		
Death (score of 1)	11 (41%)		

**Table 2:** Spearman's correlation between treatment outcome and monitored parameters.

	Parameter	Correlation coefficient	*p* value
Days 0–3 post-SAH	CRP	−0.273	0.257
	WBC	−0.433	0.063
	Hgb	0.118	0.628
	Fibrinogen	−0.286	0.248
	sRAGE	−0.177	0.454

Days 5–7 post-SAH	CRP	−0.339	0.198
	WBC	−0.333	0.207
	Hgb	0.001	0.995
	Fibrinogen	−0.484	0.057
	sRAGE	−0.302	0.254

Days 10–14 post-SAH	CRP	−0.480	0.134
	WBC	−0.086	0.800
	Hgb	0.493	0.122
	Fibrinogen	−**0.626**	**0.070**
	sRAGE	0.139	0.682

	WFNS on admission	−**0.741**	**<0.001**
	HH on admission	−**0.656**	**<0.001**
	GCS on admission	**0.688**	**<0.001**
